# Metabolic importance of adipose tissue monoacylglycerol acyltransferase 1 in mice and humans

**DOI:** 10.1194/jlr.M084947

**Published:** 2018-05-31

**Authors:** Kim H. H. Liss, Andrew J. Lutkewitte, Terri Pietka, Brian N. Finck, Michael Franczyk, Jun Yoshino, Samuel Klein, Angela M. Hall

**Affiliations:** Department of Pediatrics* Washington University School of Medicine, St. Louis, MO 63110; Medicine,† Washington University School of Medicine, St. Louis, MO 63110

**Keywords:** lipolysis, fatty acid re-esterification, lipids, adipocytes, fatty acid, lipolysis and fatty acid metabolism

## Abstract

Adipocyte triglyceride storage provides a reservoir of energy that allows the organism to survive times of nutrient scarcity, but excessive adiposity has emerged as a health problem in many areas of the world. Monoacylglycerol acyltransferase (MGAT) acylates monoacylglycerol to produce diacylglycerol; the penultimate step in triglyceride synthesis. However, little is known about MGAT activity in adipocytes, which are believed to rely primarily on another pathway for triglyceride synthesis. We show that expression of the gene that encodes MGAT1 is robustly induced during adipocyte differentiation and that its expression is suppressed in fat of genetically-obese mice and metabolically-abnormal obese human subjects. Interestingly, MGAT1 expression is also reduced in physiologic contexts where lipolysis is high. Moreover, knockdown or knockout of MGAT1 in adipocytes leads to higher rates of basal adipocyte lipolysis. Collectively, these data suggest that MGAT1 activity may play a role in regulating basal adipocyte FFA retention.

Adipose tissue is a dynamic organ that hydrolyzes and synthesizes triglycerides. Lipolysis of adipocyte triglycerides and the release of FFAs into the bloodstream provides energy for normal body organ system function during postabsorptive conditions and is critical for survival during periods of starvation. However, an increased rate of lipolysis and plasma FFA concentrations can cause multi-organ insulin resistance (1). In contrast, an increased capacity of adipocytes to produce FFAs from glucose (i.e., de novo lipogenesis) is associated with insulin sensitivity (2–4). Excessive accumulation of triglycerides in adipose tissue, which occurs in people with obesity, is associated with metabolic abnormalities and diseases, including insulin resistance, atherogenic dyslipidemia, nonalcoholic fatty liver disease, and type 2 diabetes (5). These findings underscore the importance of the balance between adipocyte triglyceride lipolysis and synthesis in regulating body fat mass and metabolic health.

For adipocytes to store lipid, FAs are sequentially esterified to a glycerol backbone to synthesize the primary form of stored energy, triglyceride. There are two major pathways for triglyceride synthesis. The canonical pathway for FA incorporation of triglyceride begins with glycerol-3-phosphate and through sequential acylation and dephosphorylation of the glycerol backbone, triglyceride is formed. Deficiencies in specific steps in this pathway are known to result in lipodystrophy and failure of adipocytes to terminally differentiate (6). The other pathway of triglyceride synthesis involves the acylation of monoacylglycerol (MG) by monoacylglycerol acyltransferase (MGAT) enzymes to form diacylglycerol (DG) (6). Relative to the glycerol-3-phosphate pathway, the role of the MGAT pathway in contributing to adipocyte lipogenesis and lipid metabolism is much less well understood. Three related MGAT-encoding genes have been identified in humans; namely, *MOGAT1*, *MOGAT2*, and *MOGAT3*, which encode MGAT1, MGAT2, and MGAT3. In mice, *Mogat1* and *Mogat2* encode MGAT1 and MGAT2, but the mouse *Mogat3* is a pseudogene and not analogous to human *MOGAT3* (7–9). Data from a series of studies have shown that MGAT2 is important for enterocyte absorption of ingested fat (7, 10–12). MGAT1 is not well-expressed in intestine, but MGAT1-mediated MGAT activity may be important in the pathogenesis of obesity-related hepatic insulin resistance (13–15). Some recent work using MGAT1 constitutive knockout mice has called into question whether MGAT1 is a significant contributor to tissue MGAT activity (16). However, acute knockdown of MGAT1 in steatotic liver reduced MGAT activity and promoted an insulin-sensitive phenotype (13). Other than in intestine and liver, the metabolic effects of MGAT enzymes have been little studied. For example, although MGAT expression and activity have been detected in adipose tissue (8, 17), its contribution to adipocyte lipid metabolism is unclear.

Because adipose tissue triglyceride synthetic rates are extremely high and *Mogat1* is known to be transcriptionally-activated by PPARγ (13, 14), the master regulator of adipogenesis, we sought to test the hypothesis that *Mogat1* is also expressed in adipocytes and regulates adipocyte lipid metabolism. We found that *Mogat1*, which encodes the MGAT1 enzyme, is robustly induced in adipocytes during the process of adipogenesis. Moreover, *Mogat1 *expression is inversely correlated with lipolytic rates, and suppression of *Mogat1* expression increases basal lipolytic activity. In addition, adipose tissue *MOGAT1* expression is greater in people with obesity who are metabolically normal than those who are metabolically abnormal. Together, these data suggest that MGAT1 is involved in both triglyceride production and lipolysis, and demonstrate increased adipose tissue *MOGAT1* expression is associated with metabolic health in people.

## MATERIALS AND METHODS

### Animals

Male mice were used in all studies. *Ob/ob* (catalog number 000632), *ob/+, db/db* (catalog number 000697), *db/+,* and C57BL/6J (catalog number 000664) mice were obtained from Jackson Laboratory (Bar Harbor, ME). All mice were maintained on LabDiet 5053. *Ob/Ob*, control littermates, and C57BL/6J mice were sacrificed at 12 weeks of age. *db/db* and control littermates were sacrificed at 14 weeks of age. All animal experiments were approved by the Animal Studies Committee of Washington University School of Medicine.

### Generation of Adn-*Mogat1*^−/−^ mice

C57BL/6 mouse embryonic stem cells containing a “knockout first” allele of *Mogat1* were obtained from the Knockout Mouse Project (Project ID CSD35789). This construct contains a targeted allele of *Mogat1* containing inserted cassettes for promoter-driven LacZ and Neo cDNAs flanked by frt sites upstream of exon 4 of the *Mogat1* gene, which is flanked by LoxP sites. Propagated ES cells were injected into developing embryos and then implanted into pseudopregnant females. Chimeric offspring were mated to establish germline transmission. Resulting heterozygous offspring were mated with C57BL/6 mice expressing Flp recombinase in a global manner (chicken α actin promoter-driven transgenic) to remove LacZ and Neo cassettes and generate mice harboring the conditional floxed allele (Fig. 5A). *Mogat1* floxed mice were then crossed with hemizygous C57BL/6 mice expressing Cre under the Adiponectin promoter to create Adn-*Mogat1* mice. Littermate mice not expressing Cre (fl/fl mice) were used as control mice in all experiments. All animal experiments were approved by the Institutional Animal Care and Use Committee of Washington University.

### Assessment of plasma metabolites

Nonesterified FFAs were measured using enzymatic assays (Wako Diagnostics, Mountain View, CA). Plasma triglyceride and cholesterol were measured using Infinity colorimetric assay kits (Thermo Fisher Scientific, St. Peters, MO). Plasma insulin was measured using an immunoassay (Singulex, Alameda, CA) by the Core Laboratory of the Diabetes Research Center at Washington University.

### Phenomaster metabolic profiling

Indirect calorimetry, energy expenditure, spontaneous activity, and food consumption were measured in Phenomaster TSE® cages as previously reported (18). Data was measured and collected at 10 min intervals for 24 h. Prior to measurements, mice were housed individually and trained for 1 day in metabolic cages before metabolic measurements were collected. Mean relative VO_2_ (expressed in ml/kg/min) and respiratory quotient (RQ; calculated as VCO_2_/VO_2_) were determined for 24 h.

### Body composition measurements

Body composition was determined using an EchoMRI 3-in-1 instrument (Echo Medical Systems). Free water mass, lean mass, and fat mass were all determined on Adn-*Mogat1* KO and WT littermates after 10 weeks on standard chow. Free water mass was less than 0.1 g for all mice.

### Adipocyte differentiation of MEFs, human preadipocytes, and 3T3-L1 fibroblasts

Primary mouse embryonic fibroblasts (MEFs) were isolated from 14-day-old mouse embryos as previously described (19). Human subcutaneous preadipoctyes were purchased from Lonza. 3T3-L1 fibroblasts were obtained from ATCC. Briefly, cells were seeded in 12-well plates and propagated to confluence. Two days postconfluence, differentiation was initiated using DMEM containing 10% FBS, 175 nM insulin, 250 nM dexamethasone, and 0.5 mM 3-isobutyl-1-methylxanthine (IBMX). Two days after initiation, the medium was replaced with a maintenance medium (DMEM containing 10% FBS and 175 nM insulin). Fresh maintenance medium was replaced every 2 days thereafter.

### siRNA-mediated *Mogat1* knockdown in differentiated 3T3-L1 adipocytes

ON-TARGETplus, SMARTpool siRNA directed against *Mogat1* (si*Mogat1*) and a nontargeting negative control (siControl) were purchased from Dharmacon (Lafayette, CO). Day 6, fully differentiated 3T3-L1 adipocytes were transfected with si*Mogat1* or siControl reagents with DarmaFECT 1 (Lafayette, CO) according to the manufacturer’s protocol. Adipocytes were treated with siRNAs for 48 h before RNA, protein, or lipolysis assays were performed.

### Lipolysis in cultured cells

Fully differentiated adipocytes were used for lipolysis studies. Adipocytes were incubated in Krebs-Ringer-bicarbonate HEPES buffer, pH 7.4) containing 4 mg/ml fatty acid free BSA and 2.5 mM glucose (with or without 1 µM isoproterenol) at 37°C for 2 h. Lipolysis was evaluated by measuring the amount of glycerol and FFAs released into the media. Glycerol was determined by a glycerol assay kit (Sigma). FFAs were quantified with a NEFA kit (Wako Diagnostics) according to the manufacturer’s instructions.

### Primary adipose tissue explants, lipolysis, and FFA re-esterification

Epididymal adipose tissue was harvested, minced under sterile conditions, and cultured for 48 h in M199 (Sigma) containing insulin (Sigma, 1.5 μg/ml) and dexamethasone (Sigma, 25 nM) at 37°C in the presence of 5% CO_2_. Immediately prior to the lipolysis assay, tissue pieces were washed to remove insulin and portioned (30–50 mg per well) into 12-well plates containing 0.25 ml of assay medium (Krebs-Ringer Buffer, pH 7.4, supplemented with fatty acid free BSA (Sigma, 4%), glucose (Sigma, 5 mM), adenosine (Sigma, 200 μM), adenosine deaminase (Roche, 1U/ml), and N6-(L-2-Phenylisopropyl)adenosine (40 nM, Sigma). To begin the lipolysis assay, wells were supplemented with an additional 250 μl of assay medium either without (basal) or with (stimulated) 0.5 μM isoproterenol. The lipolysis assay was conducted at 37°C (5% CO_2_) for 2 h. Aliquots of conditioned media were collected at 1 and 2 h and lipolysis evaluated by measuring the amount of glycerol and FFAs released into the media. Glycerol was determined by a glycerol assay kit (Sigma). FFAs were quantified with a NEFA kit (Wako Diagnostics) according to the manufacturer’s instructions.

The FFA re-esterification rate in primary epididymal adipose tissue explants was quantified using the “balance” method described by Vaughan (20). This method is based on the following formula: re-esterified FFA (nmol/mg tissue/hr) = 3 × released glycerol (nmole/mg/hr) − released FFA (nmol/mg/hr). The formula accounts for total change in both FFA and glycerol in the system (media and cell-associated). Glycerol was determined by a glycerol assay kit (Sigma). FFAs were quantified with a NEFA kit (Wako Diagnostics) according to the manufacturer’s instructions.

### MGAT enzymatic assays

MGAT assays were performed to measure initial activity rates as previously described (13). Adipocytes and fibroblasts were homogenized in 50 mM Tris-HCl (pH 7.4), 1 mM EDTA, and 0.25 M sucrose. Monoolein was dried down and reconstituted in assay buffer containing 5 mM MgCl2, 1.25 mg/ml BSA, 200 mM sucrose, 100 mM Tris-HCl (pH 7.4), 25 μM [^14^C]oleoyl-CoA, and 200 μM sn-2-oleoylglycerol. Fifty micrograms of protein were used to initiate each reaction and incubated for 5 min. The reaction was terminated by the addition of 50 ml 1% phosphoric acid. Lipids were extracted with 0.300 ml CHCl3/MeOH (2:1 vol/vol) and separated by TLC with hexane/ethyl ether/acetic acid (80:20:1 vol/vol/vol). Lipids were extracted and separated on Linear-K preadsorbent TLC plates. The spots corresponding to DG and triacylglycerol were scraped from the TLC plates and ^14^C-radioactivity was quantified by a scintillation counter.

### mRNA isolation and gene expression analyses

Total adipose tissue RNA was extracted with RNA-BEE (Iso-Tex Diagnostics, Friendswood, TX) according to the manufacturer’s instructions. First-strand cDNA synthesis was performed using ABI reagents, and quantitative real-time RT-PCR was performed using the ABI PRISM 7500 sequence detection system (Applied Biosystems, Foster City, CA) with Power SYBR green. Arbitrary units of target mRNA were corrected to the corresponding level of the 36B4 mRNA. The sequences of the primers used in these studies are found in **Table 1**.

**Table 1.** Primer sequences used for quantitative real-time PCR

Mouse Genes	Primer Sequence (5′-3′)
Atf3	For: CAG ACC CCT GGA GAT GTC AGT
	Rev: TTC TTG TTT CGA CAC TTC GCA
Ccl2	For: TTA AAA ACC TGG ATC GGA ACC AA
	Rev: GCA TTA GCT TCA GAT TTA CGG GT
Cd68	For: CCT TGA CCT GCT CTC TCT AAG G
	Rev: CTG GTA GGT TGA TTG TCG TCT G
F4/80	For: CCT GAT GGT GAG AAA CCT GA
	Rev: CCC CAG GAA ACT CCA GAT AA
Fasn	For: GTC TGG AAA GCT GAA GGA TCT C
	Rev: TGC CTC TGA ACC ACT CAC AC
Grp78	For: ACC CCG AGA ACA CGG TCT T
	Rev: GCT GCA CCG AAG GGT CAT T
Il1b	For: GTG TGT GAC GTT CCC ATT AGA C
	Rev: GTC GTT GCT TGG TTC TCC TT
Il4	For: GGTCTCAACCCCCAGCTAGT
	Rev: GCCGATGATCTCTCTCAAGTGAT
Il10	For: GGC GCT GTC ATC GAT TTC TCC CC
	Rev: AGC TCT GTC TAG GTC CTG GAG TCC
Mogat1	For: TGG TGC CAG TTT GGT TCC AG
	Rev: TGC TCT GAG GTC GGG TTC
Mogat2	For: TGG GAG CGC AGG TTA CAG A
	Rev: CAG GTG GCA TAC AGG ACA GA
Pparg	For: AAG ACC CAG CTC TAC AAC AGG C
	Rev: GCC AAC AGC TTC TCC TTC TCG G
Nos2	For: GGT TTG AAA CTT CTC AGC CAC C
	Rev: TTC TCC GTT CTC TTG CAG TTG A
Slc2a4	For: CTG TCG CTG GTT TCT CCA A
	Rev: CTG CTC TAA AAG GGA AGG TGT C
Xbp1t	For: CAG ACT ACG TGC GCC TCT GC
	Rev: CTT CTG GGT AGA CCT CTG GG
Xbp1s	For: TCT GCT GAG TCC GCA GCA GG
	Rev: CTC TAA GAC TAG AGG CTT GG
36B4	For: GCA GAC AAC GTG GGC TCC AAG CAG AT
	Rev: GGT CCT CCT TGG TGA ACA CGA AGC CC

Human Genes	Primer Sequence (5′-3′)
FASN	For: TGG AAG TCA CCT ATG AAG CCA
	Rev: ACG AGT GTC TCG GGG TCT C
MOGAT1	For: AAA GTG TGT CCT ACA TGG TAA GC
	Rev: TGA TCC TTC AGG GTT GTC AGT
PPARG	For: ATA AAG TCC TTC CCG CTG AC
	Rev: CAC CTC TTT GCT CTG CTC CT
SLC2A4	For: GCC CCA CAG AAG GTG ATT GA

### Western blot analyses

For Western blot analyses, protein extracts were obtained from tissue by using the following lysis buffer containing a protease inhibitor cocktail: 10 mM HEPES (pH 7.5), 150 mM NaCl, 1 mM EDTA, 0.1% sodium deoxycholate, and 1% Triton X-100. Antibody against MGAT1 was generated by Proteintech by using the synthetic peptide that corresponds to C-terminal amino acids 322-335 of mouse MGAT1.

### Assessments in human subjects

Subcutaneous abdominal adipose tissue samples obtained from 22 men and women with obesity (mean ± SD, BMI 40.6 ± 9.2 kg/m^2^), as part of their participation in a previous study that evaluated the metabolic effects of weight gain (3), were used to evaluate *MOGAT1* and *MOGAT2* expression. Adipose tissue samples were obtained during the basal period of a hyperinsulinemic-euglycemic clamp procedure, which was conducted in conjunction with the infusion of 6,6-^2^H_2_]glucose and [U-^13^C]palmitate tracers (Cambridge Isotope Laboratories) to assess insulin glucose rate of disappearance from plasma during basal conditions and insulin infusion and FFA rate of appearance in plasma during basal conditions, as previously described (3).

### Statistical analysis

Statistical comparisons were made using *t*-test or ANOVA where appropriate. Unless specified, all data are presented as mean ± SEM, with statistically significant difference defined as *P* < 0.05.

## RESULTS

### *Mogat1* is induced during differentiation of mouse and human adipocytes

**Fig. 1.**
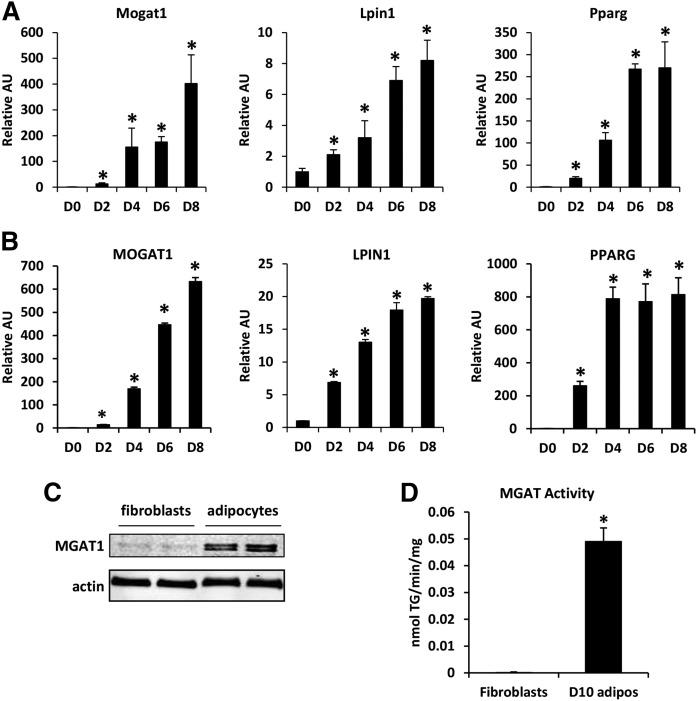
*Mogat1* is induced during differentiation of mouse and human adipocytes. A: Expression of the indicated genes in MEF-derived adipocyte precursor cells after 0, 2, 4, 6, or 8 days of culture in differentiation medium as described in the Materials and Methods. Data are presented as mean ± SEM, n = 5 per group, *P < 0.05 versus D0 fibroblasts. B: Expression of the indicated genes in human subcutaneous adipocyte precursor cells after 0, 2, 4, 6, or 8 days of culture in differentiation medium as described in the Materials and Methods. Data are presented as mean ± SEM, *P < 0.05 versus D0 fibroblasts. C: Western blot analysis for MGAT1 proteins in 3T3-L1 fibroblasts and differentiated adipocytes (D) MGAT enzymatic activity in 3T3-L1 fibroblasts or differentiated adipocytes. Data are presented as mean ± SEM, n = 5 per group, *P < 0.05 versus fibroblasts.

We evaluated the expression and activity of *Mogat1* during adipocyte differentiation. Mouse embryonic fibroblasts and human subcutaneous preadipocytes were cultured in the presence of adipocyte differentiation cocktail, and the expression of *Mogat1* and *MOGAT1* was evaluated as the cells differentiated into adipocytes. The expression of both *Mogat1* and MGAT1 was very low in both fibroblasts and preadipocytes, but increased markedly as mouse and human cells attained their differentiated phenotype (**Fig. 1A**, B). The timing of this induction coincided with an increased expression of PPARγ (Fig. 1A, B), a master regulator of adipocyte differentiation, and the expression of lipin-1, a key enzyme in the glycerol-3-phosphate pathway of triglyceride synthesis (Fig. 1A, B). Differentiation also caused an increase in the expression of *Mogat2* in the mouse adipocytes, but this increase was very small (data not shown). *MOGAT2* was undetectable in human adipocytes (data not shown). The increase in gene expression of *Mogat1* was associated with an increase in MGAT1 levels, determined by using Western blot analysis (Fig. 1C). In addition, MGAT activity was much greater in mature adipocytes than in fibroblasts (Fig. 1D), demonstrating that the increased expression of *Mogat1* results in a functional increase in enzyme activity. Together, these data indicate that the expression of MGAT1 and MGAT activity is markedly increased during adipocyte differentiation.

### *Mogat1* expression is decreased in adipose tissue of *ob/ob* and *db/db* mice

**Fig. 2.**
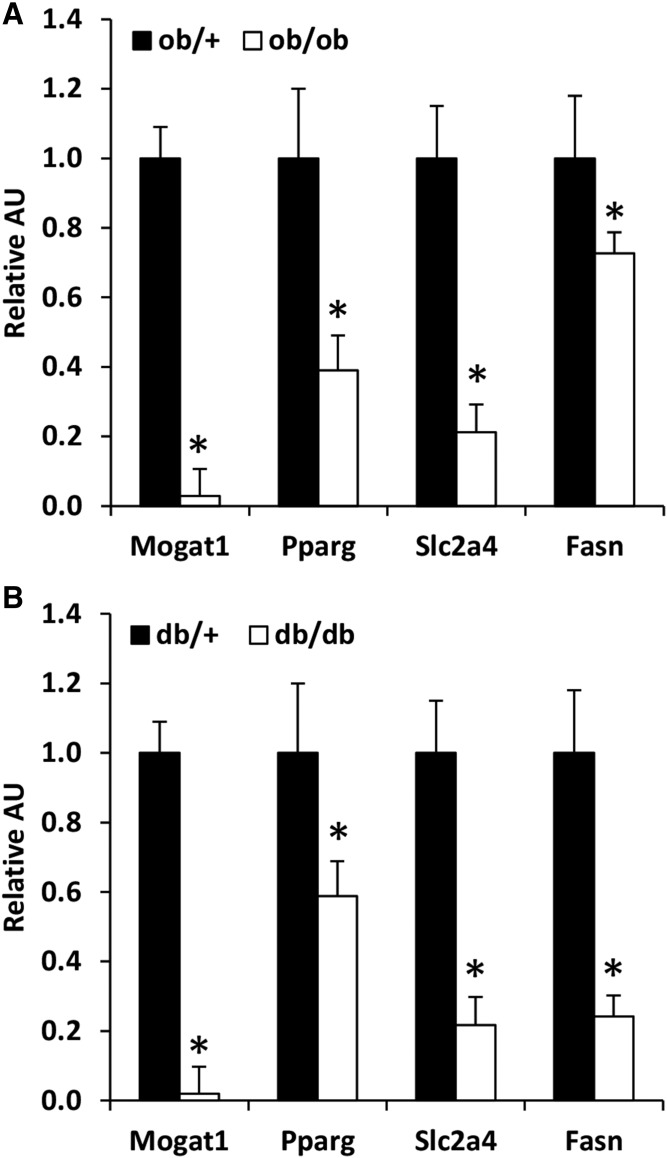
*Mogat1* expression is decreased in adipose tissue of *ob/ob* and *db/db* mice and suppressed by lipolytic stimuli. A: Expression of indicated genes in adipose tissue of *ob/+ vs ob/ob*. Data are presented as mean ± SEM, n = 6 per group, *P < 0.05 versus *ob/+ mice.* B: Expression of indicated genes in adipose tissue of *db/+ *versus* db/db* mice. Data are presented as mean ± SEM, n = 6 per group, *P < 0.05 versus *ob/+ mice.*

We sought to determine whether the expression of *Mogat1* might be regulated in adipose tissue in mouse models of metabolically abnormal obesity. Compared with *ob/+* mice, *ob/ob* mice had a much lower expression of *Mogat1* in epididymal adipose tissue (**Fig. 2A**). *Mogat1* expression was also markedly decreased in *db/db* mice compared with *db/+* mice (Fig. 2B). Consistent with a role for PPARγ in regulating *Mogat1*, the expression of *Pparg* was significantly decreased in *ob/ob* as well as *db/db* mice. Furthermore, the expression of other lipogenic genes such as fatty acid synthase (*Fasn*) and the glucose transporter, *Slc2a4* (GLUT4), was also significantly lower in the *ob/ob* and *db/db* mice than in *ob/+* and *db/+* mice.

### Adipose tissue *Mogat1* expression is suppressed by lipolytic stimuli

**Fig. 3.**
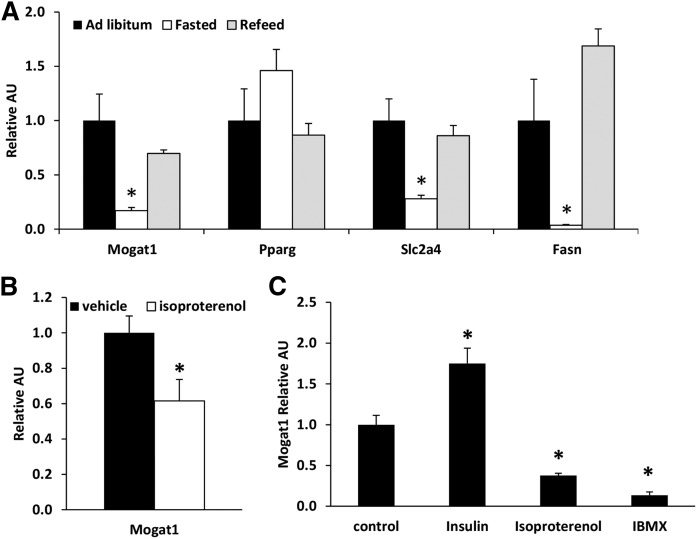
*Mogat1* expression is suppressed by lipolytic stimuli. A: Expression of indicated genes in adipose tissue of mice during fasted conditions and upon refeeding. Data are presented as mean ± SEM, n = 6 per group, *P < 0.05 versus ad libitum (fed). B: *Mogat1* expression in mice following injection of isoproterenol (25 mg/kg IP). Data are presented as mean ± SEM, n = 5 per group, *P < 0.05 versus vehicle. C: *Mogat1* expression in 3T3-L1 cells treated with insulin (100nM), isoproterenol (10μM), or isobutylmethylxanthine (IBMX, 0.5nM) for 24 h. Data are presented as mean ± SEM, n = 5 per group, *P < 0.05 versus control.

We examined the expression of *Mogat1* in mice after a prolonged (36 h) fast, which is a potent lipolytic stimulus. Fasting caused a 90% reduction in the expression of *Mogat1* in epididymal adipose tissue of C57BL/6J mice (**Fig. 3A**). Upon refeeding for 12 h after a 24 h fast, *Mogat1* expression returned to levels observed in the fed state. The expression of *Fasn* and *Slc2a4* was also suppressed in adipose tissue by fasting and restored by refeeding. However, *Pparg* expression was not regulated in parallel. We also assessed the effects of a β agonist (isoproterenol) on *Mogat1* expression. Six hours after administration of isoproterenol, *Mogat1* expression was markedly decreased in epididymal adipose tissue of mice (Fig. 3B). To examine whether these effects were direct, we treated 3T3-L1 adipocytes for 24 h with isoproterenol or IBMX, which both activate protein kinase A and lipolysis. Both isoproterenol and IBMX reduced *Mogat1* expression (Fig. 3C). In contrast, insulin treatment, which inhibits lipolytic activity, resulted in increased *Mogat1* expression (Fig. 3C). These data demonstrate a negative relationship between adipocyte lipolytic rate and *Mogat1* expression.

### *Mogat1* knockdown increases basal FFA release

**Fig. 4.**
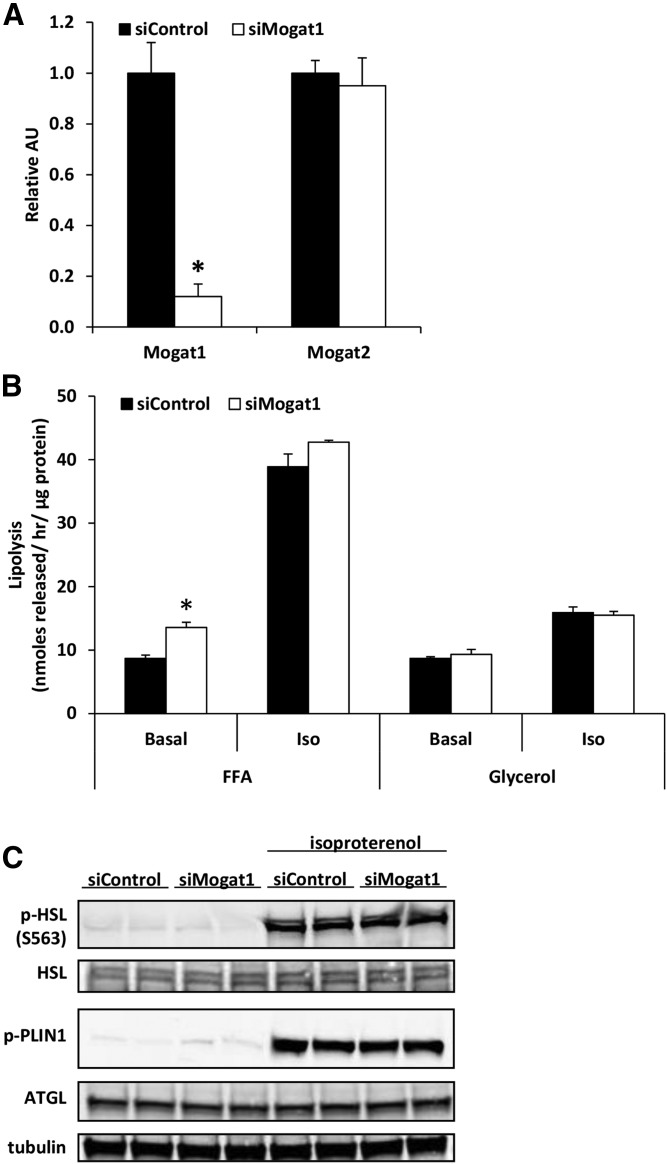
*Mogat1* knockdown in differentiated 3T3-L1 adipocytes leads to increased basal FFA release. A: *Mogat1* knockdown using siRNA in differentiated adipocytes. Data are presented as mean ± SEM, n = 5 per group, *P < 0.05 versus siControl. B: *Mogat1* knockdown in differentiated adipocytes leads to increased FFA release during basal lipolysis, but not isoproterenol stimulated lipolysis. Data are presented as mean ± SEM, n = 5 per group, *P < 0.05 versus siContol. C: Western blots for lipolytic machinery, p-HSL, total HSL, p-PLIN, ATGL, and tubulin.

To test whether suppressing *Mogat1* would affect lipolytic rates, we treated differentiated adipocytes with siRNA against *Mogat1* (si*Mogat1*) or negative control siRNA (siControl). Forty-eight hours later, *Mogat1* expression was reduced by 90% compared with siControl-treated cells, while *Mogat2* expression was unaffected by si*Mogat1* treatment (**Fig. 4A**). Differentiated 3T3-L1s (treated with the siControl or si*Mogat1* for 48 h) were serum-starved for 4 h before measuring basal and β-adrenergic stimulated-lipolysis (10 μM isoproterenol) for 1 h. A 90% reduction in expression of *Mogat1* increased adipocyte FFA release in basal conditions (Fig. 4B), but stimulated lipolytic activity was not affected. Expression and stimulated phosphorylation of the adipocyte’s major lipolytic machinery; namely, phosphorylated hormone sensitive lipase, phosphorylated perilipin 1, and adipose tissue triglyceride lipase, was not affected by knockdown of *Mogat1* (Fig. 4C), suggesting a direct effect of MGAT1 enzymatic activity on FFA re-esterification.

### Adipocyte-specific* Mogat1 *knockout mice have increased basal rates of FFA release

**Fig. 5.**
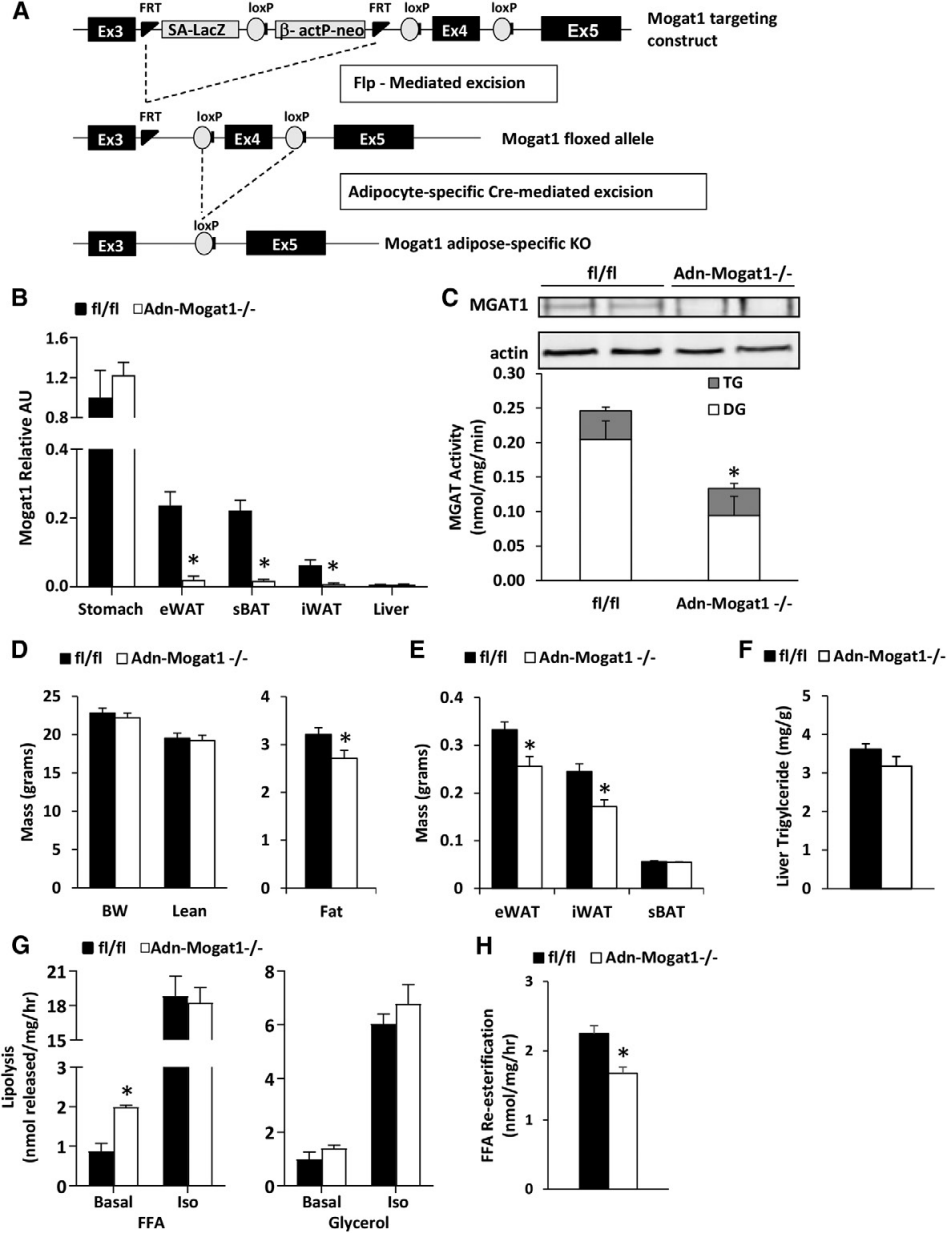
Adipocyte-specific *Mogat1* knockout mice have increased basal rates of FFA release. A: Breeding Schematic for the creation of Adn-*Mogat1*^−/−^ mice. B: *Mogat1* mRNA expression of profile of Adn-*Mogat1* mice and WT littermates. Data are presented as mean ± SEM, n = 6 per group, *P < 0.05 versus WT littermates. eWAT, epididymal adipose tissue; iWAT, inguinal adipose tissue; sBAT, subscapular brown adipose tissue. C: Western blot depicts loss of MGAT1 protein in epididymal fat pads. MGAT activity is reduced in epididymal fat pads from Adn-*Mogat1*^−/−^ mice. Data are presented as mean ± SEM, n = 5 per group, *P < 0.05 versus WT littermates. D: Body weight and composition of 10-week-old, chow-fed male Adn-*Mogat1* mice and WT littermates. Data are presented as mean ± SEM, n = 9 per group, *P < 0.05 versus WT littermates. E: Weight of eWAT, iWAT, and sBAT from 10-week-old, chow-fed Adn-*Mogat1* mice and WT littermates. Data are presented as mean ± SEM, n = 9 per group, *P < 0.05 versus WT littermates. F: Hepatic triglyceride content for 10-week-old, chow-fed Adn-*Mogat1* mice and WT littermates. Data are presented as mean ± SEM, n = 9 per group. G: Basal FFA release is increased from epididymal adipose tissue fragments from Adn-*Mogat1*^−/−^ mice under basal conditions but not isoproterenol (Iso) stimulated conditions. Data are presented as mean ± SEM, n = 6 per group, *P < 0.05 versus WT littermates. H: FFA re-esterification rate is reduced in epididymal fat pads from Adn-*Mogat1*^−/−^ mice compared with WT littermates. Data are presented as mean ± SEM, n = 5 per group, *P < 0.05 versus WT littermates.

To further confirm our in vitro findings of *Mogat1* knockdown, we generated fat-specific *Mogat1* knockout mice using Cre-LoxP mediated methods (Adn-*Mogat1*^−/−^) (**Fig. 5A**). These mice were viable and did not display an outward phenotype at baseline. Adn-*Mogat1*^−/−^ mice had significantly decreased expression of *Mogat1* in all examined fat depots, but expression levels were not altered in the stomach or the liver (Fig. 5B). Western blot analysis also demonstrated loss of MGAT1 protein in adipose tissue of the knockout mice, which was accompanied by decreased MGAT activity (Fig. 5C).

At 10 weeks old, male Adn-*Mogat1^−/−^* mice fed a standard low fat chow weighed the same as their WT littermates (Fig. 5D). To determine total body composition, mice were subjected to noninvasive EchoMRI measurements. Adn-*Mogat1^−/−^* mice and WT littermates had similar total lean mass, but Adn-*Mogat1^−/−^* mice had significantly less total fat mass compared with age-matched littermates (Fig. 5D). These measures were confirmed at euthanasia; epididymal and inguinal fat pads from the Adn-*Mogat1^−/−^* mice were significantly smaller than control littermates, while the subscapular brown fat pad was not different (Fig. 5E). Interestingly, liver triglyceride levels were not significantly affected, but trended lower, in the Adn-*Mogat1*^−/−^ mice (Fig. 5F). Blood glucose was significantly decreased in the Adn-*Mogat1^−/−^* mice after a 4 h fast (**Table 2**), whereas plasma lipids (triglycerides, FFAs, and cholesterol) were unchanged (Table 2). Interestingly, despite having smaller fat pad mass, the Adn-*Mogat1^−/−^* mice did not display significant differences in food intake, O_2_ consumption, CO_2_ production, respiratory exchange ratio, or energy expenditure during a 24-h assessment (**Table 3**).

**TABLE 2.** Plasma metabolites from 10-week-old male WT and Adn-*Mogat1*^−/−^ mice littermates after a 4 h fast

	fl/fl	Adn-*Mogat1*^−/−^	*P*
Glucose (mg/dl)	170 ± 3	155 ± 6	0.045
Insulin (ng/ml)	0.56 ± 0.05	0.69 ± 0.13	0.37
Free fatty acids (μM)	0.42 ± 0.03	0.38 ± 0.04	0.17
Triglycerides (mg/dl)	71 ± 6	67 ± 5	0.51
Cholesterol (mg/dl)	78 ± 6	83 ± 5	0.15

n = 9 per group.

**TABLE 3.** Energy balance and activity of 10-week-old male WT and Adn-*Mogat1* KO mice fed a chow diet ad libitum over 24 h

	fl/fl	Adn-*Mogat1*^−/−^	*P*
Food intake (g/day)	2.3 ± 0.1	2.2 ± 0.2	0.92
O_2_ consumption (ml/h/kg)	3143 ± 110	3286 ± 157	0.49
CO_2_ production (ml/h/kg)	2833 ± 124	2967 ± 162	0.54
Respiratory exchange ratio	0.894 ± 0.008	0.895 ± 0.006	0.92
Energy expenditure (kcal/h/kg)	15.5 ± 0.6	16.2 ± 0.8	0.50
Activity counts	295 ± 32	244 ± 43	0.42

WT, n = 4; KO, n = 4.

**Fig. 6.**
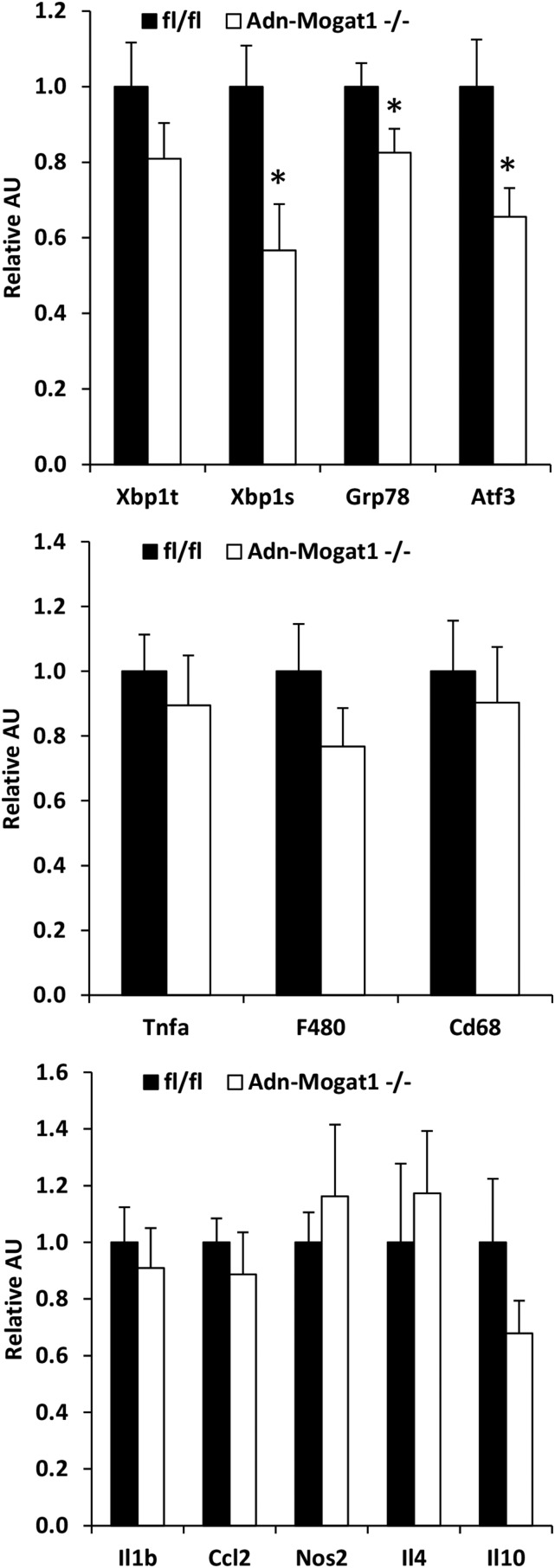
Gene expression of inflammatory markers in WT and Adn-*Mogat1*^−/−^ mice. Expression of indicated genes in epidydimal adipose tissue of obese human subjects. Data are presented as mean ± SEM, n = 6 per group, *P < 0.05.

Lipolytic rates were assessed in isolated adipose tissue explants from Adn-*Mogat1*^−/−^ mice and matched littermate controls. Although isoproterenol-stimulated adipose tissue lipolytic rate was not different between groups, the basal rate of FFA release from *Mogat1* knockout mice was significantly increased compared to control mice (Fig. 5G). Moreover, the FFA re-esterification rate was significantly decreased in the epididymal fat pads from Adn-Mogat1 mice (Fig. 5H). These data are again consistent with adipocyte MGAT1 playing a role in the recycling of FFAs and MG during conditions of low lipolytic flux. Interestingly, unlike the previous report showing that diacylglycerol acyltransferase (DGAT) deficiency led to increased endoplasmic reticulum stress due to the increase in lipolytic products (21), we found no evidence for ER stress in fat pads of Adn-*Mogat1*^−/−^ mice and the expression of some of these genes was actually reduced (**Fig. 6**).

### Adipose tissue biology in people with metabolically-normal and metabolically-abnormal obesity

**Fig. 7.**
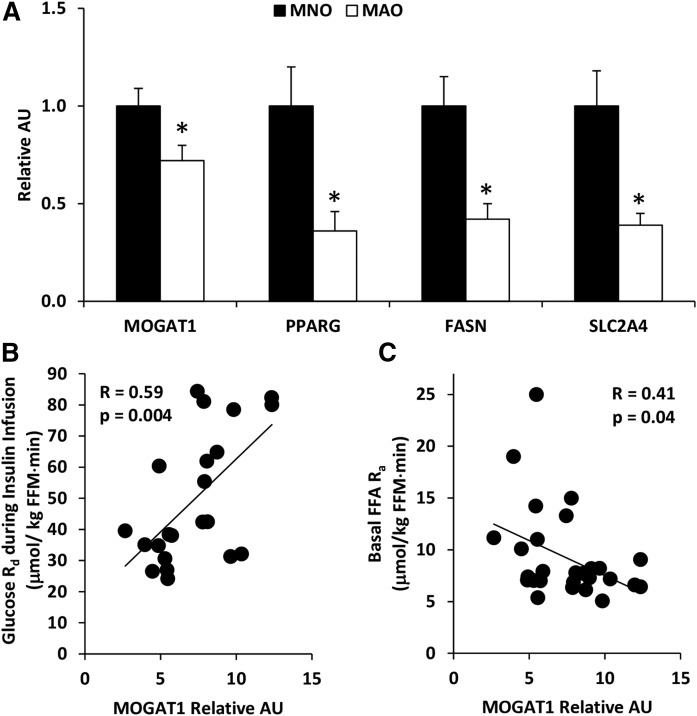
Adipose tissue biology in people with metabolically-normal and metabolically-abnormal obesity. A: Expression of indicated genes in subcutaneous adipose tissue of obese human subjects. Data are presented as mean ± SEM, *P < 0.05. B: Subcutaneous adipose tissue *MOGAT1* expression is positively correlated with the whole body glucose disposal rate during a hyperinsulinemic euglycemic clamp. C: Subcutaneous adipose tissue *MOGAT1* expression is inversely correlated with whole body lipolysis before hyperinsulinemic euglycemic clamp.

We next sought to determine whether the results from the studies conducted in mice apply to humans by evaluating subcutaneous adipose tissue *MOGAT1* expression and the relationship between adipose tissue *MOGAT1* expression and basal lipolytic activity in 22 men and women with either metabolically-normal obesity (MNO, n = 10) or metabolically-abnormal obesity (MAO, n = 12) (**Table 4**). The classification of MNO and MAO was determined by the glucose infusion rate required to maintain euglycemia during a hyperinsulinemic-euglycemic clamp procedure, based on the upper and lower tertile of values we obtained in previous studies; those with a glucose infusion rate ≥10.5 mg/kg fat-free mass/min were defined as MNO and those with a glucose infusion rate ≤6.9 mg/kg fat-free mass/min were defined as MAO. Subjects with MAO had lower subcutaneous adipose tissue expression of *MOGAT1* than those with MNO (**Fig. 7A**). Adipose tissue *MOGAT2* expression was undetectable in all subjects (data not shown). In concert with the data obtained in our rodent models of obesity and insulin resistance, adipose tissue expression of other lipogenic genes, including *PPARG*, *FASN*, and *SLC2A4*, was lower in subjects with MAO than those with MNO. In addition, adipose tissue *MOGAT1* expression was positively correlated with insulin sensitivity, assessed by the rate of glucose disposal during a hyperinsulinemic-euglycemic clamp procedure (*R* = 0.59, *P* = 0.004) (Fig. 7B), and was negatively correlated with basal adipose tissue lipolytic activity, assessed as the rate of appearance of FFA into the bloodstream determined by using a stable isotopically labeled palmitate tracer infusion (*R* = 0.41, *P* = 0.04) (Fig. 7C). Overall, these data are consistent with the concept that MAO in humans is associated with an impaired expression of adipose tissue genes encoding factors that promote triglyceride storage, and a decrease in adipose tissue *MOGAT1* expression contributes to an increase in basal adipose tissue lipolytic rate.

**TABLE 4.** Characteristics of metabolically normal and metabolically abnormal obese human subjects

	Metabolically Normal Obese	Metabolically Abnormal Obese	*P*
Sex, n (male/female)	10 (2/8)	12 (2/10)	
Age, y	48 ± 10	39 ± 12	0.062
Body mass index	35.1 ± 4.7	43.5 ± 9.3	0.015
Body fat mass, %	45 ± 7	48 ± 7	0.25
IHTG content, %	4.7 ± 3.9	11.6 ± 9.1	0.047
Glucose, mg/dl	97 ± 7	99 ± 9	0.594
Insulin, mU/L	8.9 ± 3.1	22.5 ± 7.0	0.00002
Total-cholesterol, mg/dl	170 ± 39	164 ± 26	0.681
LDL-cholesterol, mg/dl	100 ± 28	103 ± 19	0.795
HDL-cholesterol, mg/dl	48 ± 11	36 ± 6	0.010
Triglyceride, mg/dl	108 ± 58	121 ± 52	0.593

Data are mean ± SD. IHTG, intrahepatic triglyceride.

## DISCUSSION

The initial report on cloning of MGAT1 detected its expression and activity in adipocytes (8), but very little is known about the physiologic role of adipocyte MGAT1 activity. Accordingly, we conducted a series of studies in cell systems, animal models, and human subjects to explore the metabolic function of adipocyte MGAT activity and its relevance in humans. Our data demonstrate that: *1*) mice lacking *Mogat1* in adipocytes have less fat mass than WT littermates; *2*) adipose tissue expression of *Mogat1* is suppressed in response to physiologic stimuli that increase adipose tissue lipolytic activity; *3*) loss of *Mogat1* in adipocytes increases basal lipolytic rate; and *4*) adipose tissue *MOGAT1* expression was positively correlated with insulin sensitivity and negatively correlated with adipose tissue lipolytic activity in humans. Together, these data demonstrate that adipocyte MGAT activity has important physiological functions in preadipocyte differentiation and lipolysis of adipocyte triglycerides, and suggest MGAT activity may affect whole body insulin sensitivity.

Our data suggest that *Mogat1* is a novel regulator of FFA release from adipocytes. By using *Mogat1* knockdown and adipocyte specific *Mogat1* knockout mice, we demonstrated that MGAT activity decreased the release of FFAs from adipocytes during basal conditions. We postulate that MGAT activity promotes FFA re-esterification and this serves to prevent FFA release when lipolytic rates should be low. We did not identify other effects on lipolytic machinery expression or phosphorylation status after *Mogat1* knockdown, suggesting that an important function of MGAT1 is re-esterifying the FFAs back into DG to prevent their release. While basal lipolysis was increased in the setting of *Mogat1* knockdown, stimulated lipolysis was not changed. This is not completely unexpected and is consistent with our observation that there is a significant suppression of *Mogat1* expression in mouse models associated with high rates of adipose tissue lipolytic activity, including fasting, β-adrenergic receptor activation, and genetic obesity. Together, these findings suggest MGAT1 regulates FFA release during both basal and stimulated lipolytic conditions. MGAT1 activity promotes FFA reesterification, effectively minimizing FFA release during basal lipolytic conditions. However, during stimulated lipolysis, MGAT1 expression is diminished to promote maximal FFA efflux from adipocytes. In concert with this observation, it was recently reported that the final enzyme in the triglyceride synthesis pathway, DGAT1, also plays a role in FFA recycling of lipolytic products by acylating DG (21). These results suggest that these two enzymes work together to oppose lipolysis in adipocytes.

Although obesity is typically associated with a constellation of metabolic abnormalities, including insulin resistance and dyslipidemia, not all people with obesity become insulin resistant. Lipid dynamics in adipose tissue may provide an explanation for the differences in the individuals with MNO versus MAO. Indeed, it has been shown that a high capacity for both glucose uptake and de novo lipogenesis in adipose tissue is metabolically protective in people with MNO. On the other hand, individuals with obesity-related metabolic syndrome or insulin resistance have been found to have a reduced capacity in adipose tissue to take up glucose and synthesize FAs from carbohydrates. In the present study, people with MAO had significantly lower expression of *PPARG*, *FASN*, and *SLC2A4*, which is consistent with previous reports in adipose tissue of people with MAO. We also found a significant decrease in *MOGAT1* expression in individuals with MAO compared with those with MNO, suggesting that individuals with MAO also have diminished capacity for triglyceride synthesis in adipose tissue. While the sample size is small, we found *MOGAT1* expression in adipose tissue is positively correlated with insulin sensitivity and negatively correlated with basal FFA rates of release into the circulation, assessed by using the hyperinsulinemic-euglycemic clamp procedure. Furthermore, in insulin resistant people, the downregulation of *MOGAT1* in adipose tissue may contributes to decreased FFA re-esterification and increased FFA release in the blood stream. Indeed, insulin resistance is associated with increased basal lipolytic rate and the generation of FFAs (22–24), while partial inhibition of adipose tissue lipolysis can improve glucose metabolism and insulin sensitivity (25–28). However, we did not detect evidence of insulin resistance in Adn-*Mogat1*^−/−^ mice, which had less fat mass and lower blood glucose than WT controls. This will need to be examined more thoroughly and under conditions where obesity is promoted.

We found that deletion of MGAT1 in adipocytes led to a substantial reduction in MGAT enzymatic activity. These results indicate that MGAT1 protein contributes to overall MGAT activity in adipose tissue. Our findings are contradictory to those from a recent study conducted in a whole body *Mogat1* knockout mouse (16). That study found there were no deficits in tissue MGAT activity in global *Mogat1*^−/−^ mice. However, MGAT activity was undetectable in many tissues that were examined. It is possible the inability to detect any changes in MGAT activity could be due to compensation by MGAT2. Additional studies are necessary to address these discrepancies.

In summary, we demonstrate that *Mogat1* is robustly induced during adipocyte differentiation and expression of adipose tissue *Mogat1* in mice and *MOGAT1* in humans are suppressed in states of relative insulin resistance and increased lipolytic rates. In addition, our data demonstrate *MOGAT1* is likely involved in regulating FFA re-esterification into triglyceride and therefore FFA release from adipose tissue. These data collectively demonstrate a novel role for MGAT activity in regulating adipocyte lipid homeostasis with potential relevance for our understanding of the physiologic process of lipolysis and the pathophysiology of obesity.
